# Bilateral Facial Paralysis Caused by Bilateral Temporal Bone Fracture: A Case Report and a Literature Review

**DOI:** 10.1155/2015/306950

**Published:** 2015-06-14

**Authors:** Sultan Şevik Eliçora, Aykut Erdem Dinç, Sultan Bişkin, Murat Damar, Ergin Bilgin

**Affiliations:** Otorhinolaryngology Department, Zonguldak Bülent Ecevit University Faculty of Medicine, 67600 Zonguldak, Turkey

## Abstract

Bilateral facial paralysis caused by bilateral temporal bone fracture is a rare clinical entity, with seven cases reported in the literature to date. In this paper, we describe a 40-year-old male patient with bilateral facial paralysis and hearing loss that developed after an occupational accident. On physical examination, House-Brackmann (HB) facial paralysis of grade 6 was observed on the right side and HB grade 5 paralysis on the left. Upon temporal bone computed tomography (CT) examination, a fracture line exhibiting transverse progression was observed in both petrous temporal bones. Our patient underwent transmastoid facial decompression surgery of the right ear. The patient refused a left-side operation. Such patients require extensive monitoring in intensive care units because the presence of multiple injuries means that facial functions are often very difficult to evaluate. Therefore, delays may ensue in both diagnosis and treatment of bilateral facial paralysis.

## 1. Introduction

Although facial paralysis is fairly commonly encountered by ear, nose, and throat physicians, bilateral facial paralysis is very rare. The frequency of facial paralysis is 20–25 per 100,000 patients, but simultaneous bilateral facial paralysis occurs in only 1 in 5 million patients [1]. Simultaneous bilateral facial paralysis is considered present if the time elapsed from paralysis of one side to paralysis of the other side does not exceed 4 weeks [1]. A differential diagnosis of bilateral facial paralysis must consider systematic, traumatic, neuromuscular, vascular, toxic, and infectious diseases and idiopathic causes.

## 2. Case Report

**Figure 1:**
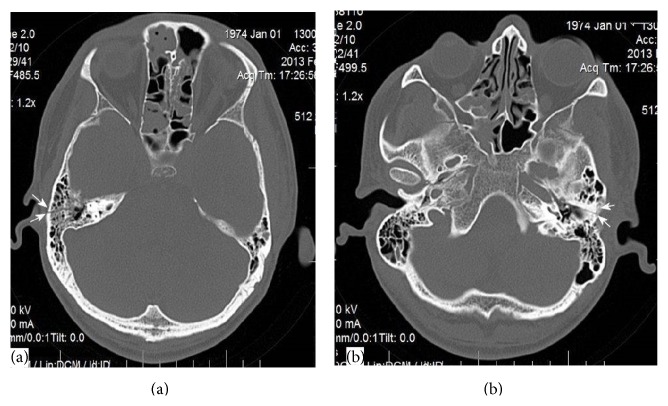
A CT scan performed on admission, showing a right transverse temporal fracture (a) and a left transverse temporal fracture (b).

**Figure 2:**
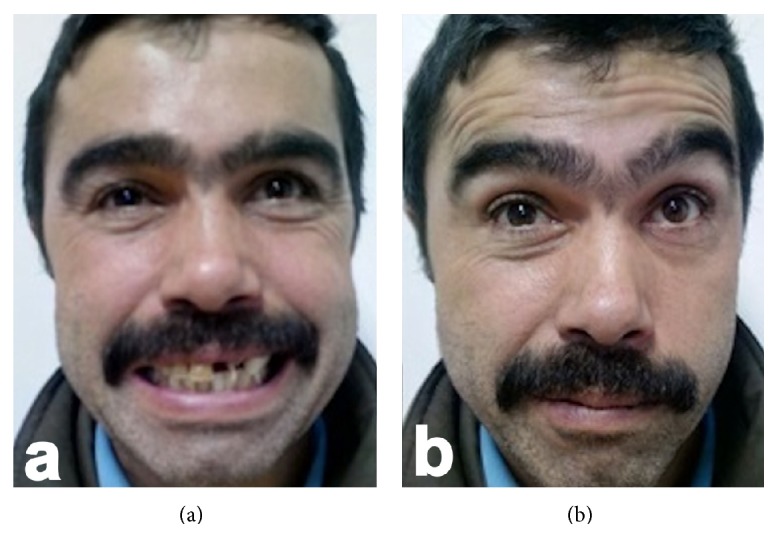
Postoperative view of the patient at the 1-year follow-up.

We present 40-year-old male patient with bilateral facial paralysis caused by blunt head trauma. The patient was followed up in our intensive care unit for 10 days, because of multiple traumas caused by the accident, and was referred to our clinic with a diagnosis of facial paralysis, hearing loss, and anosmia. He was conscious, cooperative, and oriented. On physical examination, both tympanum membranes were intact. Upon House-Brackmann (HB) classification, HB grade 6 facial paralysis was observed on the right side and HB grade 5 on the left. Upon audiometric testing, a slight sensorineural hearing loss (SNHL) was evident on the right side with advanced mixed-type hearing loss on the left. On facial maxillary computed tomography (CT) examination, multiple nondisplaced fracture lines were observed on the base of the right frontal sinus base, on the left frontal bone, on the zygomatic bone, and on both sides of the sphenoid bone. Also, a transversely progressive fracture line was evident in both petrous temporal bones (Figures 1(a) and 1(b)). As the facial paralysis had developed immediately after the accident and as total denervation was evident upon electromyography (EMG) examination, a transmastoid facial decompression operation was performed on the right side in the third week after trauma. The facial nerve was decompressed and bone spicules thereon were cleared. No postoperative complication developed. The patient refused a left-side operation. The right side was graded HB 1 and the left side HB 2 at the 1-year follow-up (Figures 2(a) and 2(b)).

## 3. Discussion

Bilateral facial paralysis caused by bitemporal bone fracture is very rare, with seven cases reported to date (Table 1).

Unlike unilateral facial paralysis, only 20% of bilateral paralysis is idiopathic [2]. The most important causes of bilateral facial paralysis are trauma, infectious diseases (infectious mononucleosis, syphilis, bilateral otitis media, herpes zoster, Lyme disease, and meningitis), neurological diseases (multiple sclerosis, neoplasms, or stroke), and other diseases, the aetiologies of which are uncertain (Guillain-Barre syndrome, sarcoidosis, Melkersson-Rosenthal syndrome, and leukaemia) [3].

Trauma causes 5% of all facial paralysis [4], and 3% of cases are associated with temporal bone fractures [5], which can be divided into longitudinal, transverse, and mixed fractures. The fracture lines of transverse fractures are vertical, running through the squamous petrous bone; these constitute 10% of all temporal bone fractures [5]. A total of 30–50% of patients with transverse fractures present with facial paralysis and SNHL [5]. Such facial paralysis is rather serious, and the prognosis is very poor [2]. In longitudinal fractures, the fracture lines lie parallel to the long axis of the temporal bone. Such fractures constitute 90% of all temporal bone fractures and, in 10–25% of cases, are accompanied by facial paralysis [6]. Mixed fractures occur at rates of 0–20%. In our patient, a fracture line exhibiting transverse progression was observed in both temporal bones, and facial paralysis was accompanied by SNHL in the right ear and a mixed type of hearing loss in the left ear.

Electrodiagnostic evaluation is useful to predict the prognoses of patients with traumatic facial paralysis. The technique explores the extent of facial nerve activity, and denervation of over 90% has been identified when electroneuronography (ENoG) was performed within 6 days of the trauma. EMG and high-resolution CT aid surgical decision-making for patients who are diagnosed late. In the present case EMG test revealed that there were bilateral fibrillation potentials and no response after stimulation, which is a typical pattern of a complete degeneration of the nerve. The prognoses of such patients depend on the extent of nerve damage. Such patients may be divided into four groups: those suffering from neuropraxia, those suffering from axonotmesis, those suffering from neurotmesis, and those suffering from nerve injuries. In patients with neuropraxia, myelin sheath damage is evident but the axon is robust. Recovery is assured. Apart from damage to the myelin sheath, patients with axonotmesis also suffer from axonal damage. Distal axons undergo Wallerian degeneration after 7 days of injury, followed by axonal regeneration from intact nervous tissue. Loss of nerve sheath continuity is evident in patients with neurotmesis. Regeneration commences but recovery is never complete. If nerve incision is evident, it is necessary to place a nerve graft in the area in which the nerve incision has caused neural function to be lost. In our case, we found that facial nerve integrity was in fact complete but that bone spicules had compressed the facial nerve.

The choice of surgical approach is important when treating patients with facial paralysis. The middle cranial fossa approach is used to treat not only longitudinal fractures, but also mixed and transverse fractures, combined with a transmastoid exploration technique [9]. We employed the latter technique in our patient; we followed the fracture line and cleared bone spicules thereon.

The timing of surgery is as important as the form of facial paralysis. Fisch stated that an intraneural haematoma might compress a nerve in those with delayed paralysis and recommended urgent exploration. Watchful waiting for 3-4 weeks was suggested for patients with early-stage paralysis. This was because the nerve was readily visualised (oedema was present) and because haematoma regression was often evident in those with early-stage paralysis [12]. The present case was diagnosed as complete bilateral facial paralysis at the first day of trauma. He was operated upon in the third week of trauma after 10-day follow-up in the intensive care unit.

In patients with facial paralysis, it is important to consider corneal irritation and ulceration. The use of synthetic tears and eye closure at night reduce the risk of keratitis [3].

Traumatic bilateral facial paralysis is very rare and is often accompanied by serious fractures of the skull base. As patients with such fractures exhibit high levels of mortality, death may occur before diagnosis. This may explain why so few cases have been reported.

Early diagnosis and treatment are vital in patients with bilateral facial paralysis. However, such patients may experience long stays in intensive care units, associated with delays in diagnosis, because facial asymmetry cannot be evaluated if the facial paralysis is bilateral. In such situations, it may be helpful to note that a patient cannot close his or her eyes completely and that the eye globes move up or out (the Bell phenomenon) when she or he is asked to close the eyes [3].

## Figures and Tables

**Table 1:** Bilateral facial paralysis depending on bilateral temporal bone fractures described in literature.

Author	Age	Gender	Occurrence	Start time of facial paralysis (date)	Concomitant cranial nerve deficit	Radiologic findings
Chitkara et al. (2002) [7]	20	M	Car accident	Right: 2 Left: 2	—	Right: petrous fracture Left: petrous fracture

Lee and Halcrow (2002) [8]	29	M	Motorbike accident	Right: 5 Left: immediate	—	Right: petrous fracture Left: oblique petrous fracture

Li et al. (2004) [5]	16	M	Car accident	Right: 7 Left: 7	—	Right: longitudinal petrous fracture Left: transverse petrous fracture

Ulug and Ulubil (2005) [9]	26	M	Motorbike accident	Right: immediate Left: immediate	VI (right)	Right: longitudinal petrous fracture Left: longitudinal petrous fracture

Roth et al. (2007) [10]	27	M	Blunt trauma	Right: 9 Left: 9	III (right, left)	Right: longitudinal petrous fracture Left: transverse petrous fracture

Saidha et al. (2010) [11]	45	M	Fall	Right: immediate Left: immediate	—	Right: transverse petrous fracture Left: transverse petrous fracture

Undabeitia et al. (2013) [3]	38	M	Fall	Right: 4-5 days Left: 4-5 days	—	Right: longitudinal petrous fracture Left: transverse petrous fracture
